# Preoperative clinical characteristics scoring system for differentiating uterine leiomyosarcoma from fibroid

**DOI:** 10.1186/s12885-020-07003-z

**Published:** 2020-06-03

**Authors:** Guorui Zhang, Xin Yu, Lan Zhu, Qingbo Fan, Honghui Shi, Jinghe Lang

**Affiliations:** Department of Obstetrics and Gynecology, Peking Union Medical College Hospital, Peking Union Medical College, Chinese Academy of Medical Sciences, No. 1 Shuaifuyuan, Wangfujing, Dongcheng District, Beijing, 100730 People’s Republic of China

**Keywords:** Uterine leiomyosarcoma, Fibroid, Diagnosis

## Abstract

**Background:**

Morcellation may lead to intraperitoneal spread of tumor cells, thus making prognosis of undiagnosed uterine leiomyosarcoma (ULMS) worse. However, preoperative diagnosis of ULMS remains challenging. This study aimed to design a preoperative clinical characteristics scoring system for differentiating ULMS from uterine fibroid.

**Methods:**

This study enrolled 45 ULMS patients and 180 uterine fibroid patients in Peking Union Medical College Hospital from January 2013 to December 2018.

**Results:**

The incidence of occult ULMS was 0.59% (95% CI, 0.39–0.71%). Age ≥ 40 years old (OR 2.826, 95%CI 1.326–5.461), tumor size ≥7 cm (OR 6.930, 95% CI 2.872–16.724), neutrophil-to-lymphocyte ratio (NLR) ≥ 2.8 (OR 3.032, 95%CI 1.288–7.13), number of platelet ≥298 × 10^9^/L (OR 3.688, 95%CI 1.452–9.266) and lactate dehydrogenase (LDH) ≥ 193 U/L (OR 6.479, 95%CI 2.658–15.792) were independent predictors of ULMS. A preoperative clinical characteristics scoring system was designed based on OR values, with a total score of 7 points. Tumor size ≥7 cm, LDH ≥ 193 U/L were assigned 2 points, while age ≥ 40 years old, NLR ≥ 2.8 and number of platelet ≥298 × 10^9^/L were assigned 1 point. Score ≥ 4 points was a useful predictor in diagnosing ULMS from fibroid (sensitivity 0.800, specificity 0.778).

**Conclusions:**

The incidence of occult ULMS was low. Age ≥ 40 years old, tumor size ≥7 cm, LDH ≥ 193 U/L, NLR ≥ 2.8 and number of platelet ≥298 × 10^9^/L were independent predictors of ULMS. The preoperative clinical characteristics scoring system could be helpful in preoperative diagnosis of occult ULMS.

## Background

Uterine fibroid is the most common benign tumor in gynecology, affecting 40–60% of all reproductive women, and is the main cause of hysterectomy worldwide. Minimally invasive surgeries, including myomectomy and hysterectomy, have advantages of low complication rate, mild pain and quick recovery. Morcellation provides possibility of minimally invasive surgery in giant uterine fibroid. Uterine leiomyosarcoma (ULMS) is a rare uterine malignant tumor originating from smooth muscle cell of uterine myometrium, with high malignancy and poor prognosis. The 5-year overall survival rate for International Federation of Gynecology and Obstetrics (FIGO) stage III-IV ULMS is approximately 25–33% [1]. Morcellation may lead to intraperitoneal spread of tumor cells, thus making prognosis of undiagnosed ULMS patients worse. Compared to open surgery group, incidence of peritoneal dissemination in stage I and II ULMS increases (44% vs. 12.9%) in morcellation group [2]. Besides, intraperitoneal recurrence rate of ULMS is higher in morcellation group than open surgery group (72.2% vs 41.2%) [3], and 3-year overall survival rate is lower (64% vs. 73%) [4]. Therefore, preoperative diagnosis of ULMS, which can reduce the risk of tumor spread caused by morcellation, is of great significance.

However, it remains challenging in differentiating ULMS from uterine fibroid preoperatively. Ultrasonography is a preferred imaging modality, and ULMS characterizes unclear boundary, low resistance and high velocity blood flow. Unfortunately, ultrasonography has no definite diagnostic value due to great heterogeneity of ULMS. MRI performs slightly better, but does not facilitate a diagnosis with certainty. Furthermore, the great cost and limited access reduce its indications for uterine tumor.

Therefore, the objective of this study is to design a preoperative clinical characteristics scoring system for differentiating ULMS from uterine fibroid.

## Methods

### Patients and study design

This study retrospectively enrolled 45 ULMS patients receiving surgery in Peking Union Medical College Hospital, a complicated and severe cases referral center in China, from January 2013 to December 2018. The inclusion criteria were: a) postoperative pathological conformation of ULMS; b) initial treatment (no surgical intervention was performed previously); c) no evidence of tumor dissemination before surgery. 180 patients with uterine fibroid were enrolled in the control group, who were matched according to time of surgery (± 1 year) and surgeons, at a ratio of 1:4. Patients with inflammatory disease or other malignant tumors were excluded.

### Data collection

Patients’ clinical data, ultrasonographic features, laboratory tests and surgical methods in ULMS group and control group were retrospectively retrieved. Ultrasonographic features included the largest diameter of uterus, number of fibroid and the largest diameter of fibroid. Laboratory tests included the number of neutrophil, lymphocyte, platelet, neutrophil to lymphocyte ratio (NLR), and serum lactate dehydrogenase (LDH) level. Ultrasonography and laboratory tests were performed within one month prior to surgery. This study was approved by the Institutional Review Board of Peking Union Medical College Hospital and written informed consents were obtained.

### Statistical analysis

SPSS20.0 was used for data analysis. Continuous variables were compared by independent sample student T test, and categorized variables by Chi-square test or Fisher exact test. Multiple logistic regression analysis was performed to define risk factors of ULMS, which formed the scoring system. The scoring system was evaluated by ROC curve with sensitivity and specificity. A *p*-value of < 0.05 was considered as statistical significance.

## Results

### Incidence of occult ULMS

From January 2013 to December 2018, 50 pathologically diagnosed ULMS patients received initial treatments in Peking Union Medical College Hospital. Of all, 5 cases were suspected as malignancy prior to operation due to evidence of tumor dissemination, and 45 cases were defined as occult ULMS. During the same period, 8169 uterine fibroid patients underwent myomectomies or hysterectomies (transabdominal, laparoscopic, transvaginal or hysteroscopic). The incidence of occult ULMS was 0.59% (95% CI, 0.39–0.71%).

### Patients characteristics

45 patients in ULMS group and 180 patients in control group were enrolled in this study. The preoperative clinical characteristics of the two groups were shown in Table 1. Patients in ULMS group were older (47.0 vs 41.2 years old, *P* < 0.001), with a higher postmenopausal rate (20.0 vs 5.0%, *P* = 0.003). There were no significant differences in BMI, clinical manifestations (including abnormal vaginal bleeding and compression symptoms) between the two groups. Ultrasonographic manifestations showed that the maximum diameter of uterus (9.6 vs 8.0 cm, *P* = 0.009) and the largest diameter of fibroids (7.8 vs 6.2 cm, *P* < 0.001) in ULMS group were significantly larger than control group. As to laboratory tests, LDH level (212.3 vs 169.1 U/L, *P* < 0.001), number of neutrophil (5.4 vs 3.5 × 10^9^/L, *P* < 0.001) and platelet (323.4 vs 272.1 × 10^9^/L, *P* = 0.005), NLR (3.3 vs 2.3, *P* = 0.007) in ULMS group were significantly higher than control group,. There were no significant differences in number of lymphocyte, hematocrit, CA125 and CA199 level.

**Table 1:** The preoperative clinical characteristics of ULSM group and control group

	ULMS group (*N* = 45)	Control group (*N* = 180)	P
Age, yrs	47.0 ± 9.7	41.2 ± 7.7	<0.001
Postmenopausal	9 (20.0)	9 (5.0)	0.003
Gravidity	2.2 ± 1.9	1.9 ± 1.4	0.169
Parity	1.1 ± 0.6	0.8 ± 0.6	0.008
BMI, kg/m^2^	23.0 ± 2.9	22.8 ± 3.5	0.801
Manifestations	27 (60.0)	106 (58.9)	0.892
Abnormal uterine bleeding	20 (44.4)	85 (47.2)	0.738
Compression symptoms	4 (8.9)	23 (12.8)	0.612
Overall uterine size, cm	9.6 ± 2.6	8.0 ± 2.4	0.009
Myoma size, cm	7.8 ± 2.3	6.2 ± 2.5	<0.001
≥8 cm	24	32	<0.001
Solitary presumed myoma	19	72	0.786
CA125, IU/L, in 140 cases	30.0 ± 39.6	23.9 ± 17.6	0.218
CA199, IU/L, in 72 cases	9.8 ± 8.9	20.2 ± 31.0	0.136
Neutrophil, × 10^9^/L	5.4 ± 3.2	3.5 ± 1.2	<0.001
Lymphocyte, × 10^9^/L	1.8 ± 0.6	2.5 ± 1.2	0.669
NLR	3.3 ± 2.5	2.3 ± 1.1	0.007
HCT, %	37.0 ± 5.3	37.6 ± 3.8	0.446
Platelet, ×10^9^/L	323.4 ± 112.2	272.1 ± 71.4	0.005
LDH, U/L	212.3 ± 57.2	169.1 ± 27.0	<0.001
Initial surgery			<0.001
Total hysterectomy	33	57	
Myomectomy	12	123	

Data was given as mean ± SD or n (%)

*ULSM* uterine leiomyosarcoma, *BMI* body mass index, *NLR* neutrophil-to-lymphocyte ratio, *LDH*, lactate dehydrogenase, *HCT* hematocrit

### Risk factors of ULMS

To differentiate ULMS from uterine fibroid, ROC curves of single factor, including age, the largest diameter of fibroid, LDH level, NLR and number of platelet were drawn respectively. The cutoff value of each factor was determined according to ROC curves, with age ≥ 40 years old, tumor size ≥7 cm, LDH ≥ 193 U/L, NLR ≥ 2.8 and number of platelet ≥298 × 10^9^/L.

Multivariate logistic regression analysis was performed to evaluate risk factors of ULMS. And Table 2 showed that age ≥ 40 years old (OR 2.826, 95%CI 1.326–5.461), tumor size ≥7 cm (OR 6.930, 95% CI 2.872–16.724), NLR ≥ 2.8 (OR 3.032, 95%CI 1.288–7.13), number of platelet ≥298 × 10^9^/L (OR 3.688, 95%CI 1.452–9.266) and LDH ≥ 193 U/L (OR 6.479, 95%CI 2.658–15.792), were independent predictors of ULMS.

**Table 2:** Risk factors of ULMS were evaluated by multivariate logistic regression analysis

Factor	*P* value	OR	95%CI
Age ≥ 40 yrs	0.044	2.826	1.326–5.461
Postmenopausal	0.122	2.642	0.770–9.068
Tumor size ≥7 cm	<0.001	6.930	2.872–16.724
NLR ≥ 2.8	0.011	3.032	1.288–7.139
Platelet ≥298 × 109/L	0.006	3.688	1.452–9.266
LDH ≥ 193 U/L	<0.001	6.479	2.658–15.792

*ULSM* uterine leiomyosarcoma, *NLR* neutrophil-to-lymphocyte ratio, *LDH* lactate dehydrogenase, *OR* odd ratio, *CI* confidential interval

### A preoperative clinical characteristics scoring system

**Fig. 1:**
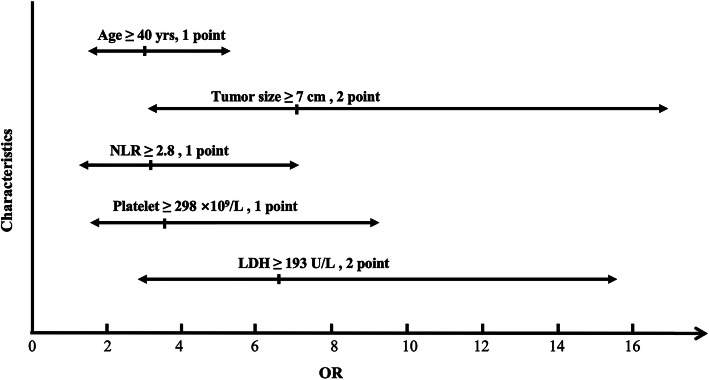
The preoperative clinical characteristics scoring system was designed based on OR values of risk factors. Factors including tumor size ≥7 cm, LDH ≥ 193 U/L were assigned 2 points, while factors including age ≥ 40 years old, NLR ≥ 2.8 and number of platelet ≥298 × 10^9^/L were assigned 1 point. OR, odd ratio; LDH, actate dehydrogenase; NLR, neutrophil-to-lymphocyte ratio

A preoperative clinical characteristics scoring system was designed based on OR values of the above factors. As shown in Table 3 and Fig. 1, tumor size ≥7 cm, LDH ≥ 193 U/L were assigned 2 points, while age ≥ 40 years old, NLR ≥ 2.8 and number of platelet ≥298 × 10^9^/L were assigned 1 point. The total score was 7 points, and each patient was scored according to the scoring system.

**Table 3:** A preoperative clinical characteristics scoring system for differentiating ULMS from fibroid

Score	0	1	2
Age, yrs	<40	≥ 40	–
Myoma size, cm	<7	–	≥ 7
NLR	<2.8	≥ 2.8	–
LDH, U/L	<193	–	≥ 193
Platelet, ×109/L	<298	≥ 298	–

*ULSM* uterine leiomyosarcoma, *NLR* neutrophil-to-lymphocyte ratio, *LDH* lactate dehydrogenase

**Fig. 2:**
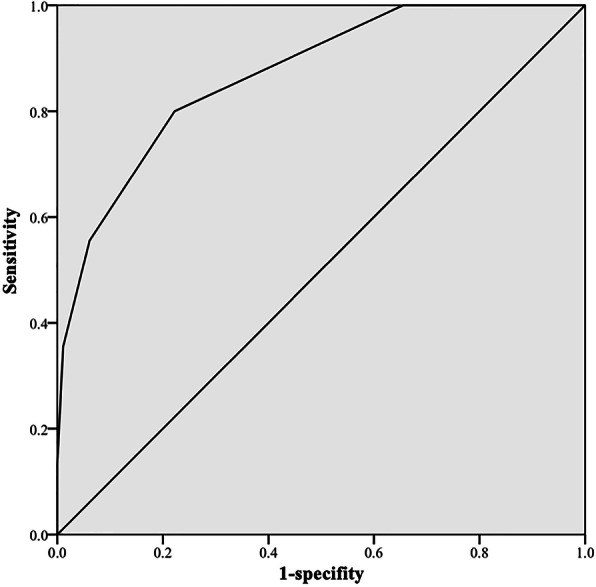
ROC curve of the scoring system was shown. The optimal cutoff value was established at 4 points, with sensitivity of 0.800 and specificity of 0.778

Scores of ULMS group and control group, together with sensitivities and specificities of different scores, were shown in Table 4. ROC curve of the scoring system was shown in Fig. 2. According to ROC curve, the optimal cutoff value was established at 4 points, with sensitivity of 0.800 and specificity of 0.778 in diagnosing ULMS from fibroid.

**Table 4:** Scores of ULMS group and control group, and sensitivity, specificity and accuracy of different scores

Scoring	≥0	≥1	≥2	≥3	≥4	≥5	≥6	≥7
ULMS group	45	45	45	39	36	25	16	6
Control group	180	156	118	66	40	11	2	0
Total	225	201	163	105	76	36	18	6
Sensitivity	1	1	1	0.867	0.800	0.556	0.356	0.133
Specificity	0	0.133	0.344	0.633	0.778	0.939	0.989	1
Accuracy(%)	20.0	30.7	47.6	68.0	78.2	86.2	86.2	82.6

*ULSM* uterine leiomyosarcoma

### Exploring efficacy of the scoring system among patients under 40 years old

Patients under 40 years old with suspected uterine fibroid were more likely to retain uterus and received myomectomy due to fertility demands. Therefore, it was of greater clinical significance to distinguish ULMS from fibroid among patients under 40. In this study, there were 85 patients under 40, including 7 ULMS patients and 78 fibroid patients. The cutoff value of each risk factor was set according to ROC curves, with tumor size ≥7 cm, LDH ≥ 189 U/ L, NLR ≥ 1.69 and number of platelet ≥251 × 10^9^ / L. Accordingly the scoring system was revised as: tumor size ≥7 cm and LDH ≥ 189 U/L assigned 2 points, while NLR ≥ 1.69 and number of platelet ≥251 × 10^9^ / L assigned 1 point. The total score was 6 points. 5 out of 7 ULMS patients and 9 out of 78 patients were scored ≥3, with sensitivity of 0.714 and specificity of 0.885. Since the number of ULMS patients under 40 was limited in this study, larger sample researches were needed to validate the finding in future.

## Discussion

In this study, the incidence of occult ULMS was 0.59%. Multivariate logistic regression analysis showed that age ≥ 40 years old, tumor size ≥7 cm, LDH ≥ 193 U/L, NLR ≥ 2.8 and number of platelet ≥298 × 10^9^/L were independent predictors of ULMS. The preoperative clinical characteristic scoring system was designed according to the OR values of different risk factors, and scores ≥4 points was an useful predictor in diagnosing ULMS from fibroid, with sensitivity of 0.800 and specificity of 0.778.

Uterine sarcoma was a rare interstitial tissue derived malignant tumor, accounting for 3–7% of uterine malignant tumors. ULMS was the most common type, accounting for 63% of uterine sarcoma [5]. Clinical manifestations of ULMS were nonspecific, and most were found accidentally after hysterectomy or myomectomy. The incidence of occult ULMS was 0.0–0.64% (median 0.22%) among presumed leiomyoma [6]. The study with the largest sample size till now reported that, rate of occult sarcoma was 0.36% (1/278) among 34,728 hysterectomies performed for presumed fibroids, and the rate of occult ULMS was 0.23% (1/429) [3]. Incidence of ULMS in this study was 0.59%, consistent with previous reports.

Age was a predictor of ULMS. A multicenter retrospective study reported 221 patients of ULMS and 81 patients of endometrial stromal sarcoma were diagnosed at an average age of 55 years old, and 59% were postmenopausal [7]. Hosh et al. found that the age-adjusted incidence rate for patients aged 50 years or older was higher than younger patients (6.4/10 vs 1.5/10, *P* < 0.0001), based on Surveillance, Epidemiology, and End Results (SEER) database [8]. In this study, patients in ULMS group were older (47.0 vs 41.2 years old, *P* < 0.001) than control group, and age ≥ 40 years old (OR 2.826, 95%CI 1.326–5.461) was an independent predictor of ULMS.

Tumor size might be a predictor of ULMS. In 2017, the European Society of Gastrointestinal Endoscopy (ESGE) confirmed that certain signs could cause suspicion of sarcoma, including a lesion exceeding 8 cm [9]. In a study of 15 ULMS patients, tumor size > 7 cm (adjusted-OR 0.973; 95% CI, 0.75–1.26; *P* = 0.08) might be a predictor of ULMS [10]. Another study involving 31 uterine sarcoma patients showed that large tumor size (> 8.0 cm) was an independent risk factor (*P* = 0.048) [11]. Results in this study also indicated that tumor size of ULMS was significantly larger than fibroid (7.8 vs 6.2 cm, *P* < 0.001), and tumor size ≥7 cm was an independent predictor of ULMS (OR 6.930, 95% CI 2.872–16.724). However, some studies had opposite results. Chen’s study with 66 cases of uterine sarcoma showed no difference in size between uterine sarcoma and fibroid (9.6 vs 8.5 cm, *P* = 0.40) [12].

Recent studies had confirmed that NLR might a markers for diagnosis and prognosis of malignant tumors. It was generally believed that inflammation was involved in the process of tumor proliferation, angiogenesis, metastasis and therapeutic response. The concentration of inflammatory cells in tumor microenvironment was high. Some secondary hematological changes might occur in tumor patients, including lymphocyte decrease, neutrophil and platelet elevation. In recent years, studies had found that NLR and platelet to lymphocyte ratio (PLR) might be markers for diagnosis and prognosis of colorectal cancer, ovarian epithelial cancer, liver cancer, pleural malignant mesothelioma, non-small cell lung cancer and breast cancer [13, 14]. Moreover, a meta-analysis enrolling 2820 patients in 14 studies showed that NLR before treatment was associated with the prognosis of soft tissue sarcoma [15]. A few studies focused on the diagnostic value of NLR in uterine sarcoma. Kim compared NLR with serum CA125 as preoperative diagnostic markers for uterine sarcoma, and NLR (> 2.12) was found to be more accurate (sensitivity, 74.5%; specificity, 70.3%) [16]. A study of 31 cases of ULMS, endometrial stromal sarcoma and undifferentiated sarcoma showed that NLR > 2.1 (*P* = 0.041) was an independent predictor of uterine sarcoma [11]. In this study enrolling 45 ULMS patients, number of neutrophil (5.4 vs 3.5 × 10^9^/L, *P* < 0.001), NLR (3.3 vs 2.3, *P* = 0.007) and number of platelet (323.4 vs 272.1 × 10^9^/L, *P* = 0.005) increased significantly; and NLR ≥ 2.8 (OR 3.032, 95%CI 1.288–7.139) and number of platelet ≥298 × 10^9^/L (OR 3.688, 95%CI 1.452–9.266) were independent predictors of ULMS.

LDH level was one of the markers in cancer diagnosis and prognosis. Metabolism of cancer cells was characterized by increased glucose uptake and lactic acid production. LDH reversibly catalyzed the conversion of pyruvate to lactate or lactate to pyruvate. Elevated LDH level was a negative prognostic biomarker not only because it was a key enzyme involved in cancer metabolism, but also it allowed neoplastic cells to suppress and evade the immune system by altering tumor microenvironment [17]. An immunohistochemical stain study of 50 uterine sarcoma and 26 fibroids revealed that the positivity rates for LDH-A and LDH-D were significantly higher in patients with uterine sarcoma (*P* < 0.05) [18]. Nagai summarized 15 cases of uterine sarcoma and showed that LDH > 279 U/L was an independent predictor in the diagnosis of uterine sarcoma [19]. LDH was also used in the diagnosis of other gynecological tumors. A study involving 20 cases found that LDH might be one of the diagnostic markers for ovarian epithelial cancer (AUC = 0.77) [20]. In addition, LDH was considered as a prognostic factor for sarcoma. LDH level before treatment was an effective marker of the prognosis of Ewing’s sarcoma [21]. In this study, LDH level in ULMS group was higher (212.3 vs 169.1 U/L, *P* < 0.001) than control, and LDH ≥ 193 U/L (OR 6.479, 95%CI 2.658–15.792) was an independent predictor of ULMS.

Due to the limited diagnostic value of single factor in ULMS, a few researchers had explored scoring systems incorporating multiple factors to increase diagnostic value. Nagai enrolled 9 patients with ULMS, 3 patients with adenosarcoma and 3 patients with endometrial stromal sarcoma [22]. A revised pre-operative sarcoma scoring system was designed, including age, serum LDH level and cytological finding, with a maximum score of 10 points and an optimal cut-off value of 4 points. The accuracy, positive predictive value, and negative predictive value were 93.7, 92.3 and 94.0%, respectively. Since adenosarcoma and endometrial stromal sarcoma were more easily detected by cytological examinations than ULMS, the results of this study were perhaps not suitable for ULMS. In Cho’s study, sarcoma index was calculated by summing the number of risk factors. Higher sarcoma index was associated with increased risk of uterine sarcoma (0, 13.6%; 1, 21.7%; 2, 62.5%; 3, 100%) [11]. In this study, according to the OR values of different risk factors, a preoperative clinical characteristics scoring system was designed, including factors of age ≥ 40 years old, tumor size ≥7 cm, LDH ≥ 193 U/L, NLR ≥ 2.8 and number of platelet ≥298 × 10^9^/L, with optimal cutoff value 4 points, sensitivity 0.800 and specificity 0.778.

As far as we knew, this was the largest sample study focusing on preoperative diagnosis of ULMS. The scoring system promoted to preoperative diagnosis of ULMS and reduced risk of dissemination of ULMS caused by morcellation. The main limitation of this study was it was a single center based retrospective study. And multicenter studies with larger sample size were needed to confirm the relevant conclusions.

## Conclusion

The incidence of occult ULMS was low and preoperative diagnosis was difficult. Age ≥ 40 years old, tumor size ≥7 cm, LDH ≥ 193 U/L, NLR ≥ 2.8 and number of platelet ≥298 × 10^9^/L were independent predictors of ULMS. The ULMS preoperative clinical characteristics scoring system could be helpful in preoperative diagnosis of occult ULMS.

## Data Availability

The datasets used in the current study available from the corresponding author on reasonable request.

## Abbreviations

ULMS: Uterine leiomyosarcoma
NLR: Neutrophil to lymphocyte ratio
LDH: Lactate dehydrogenase
